# A Comparative Study of Intravenous Ondansetron and Pericardium 6 Acupressure Point Stimulation for the Reduction of Postoperative Nausea and Vomiting

**DOI:** 10.7759/cureus.84664

**Published:** 2025-05-23

**Authors:** Sanjay Kumar Sureen, Faris Dawood Alaswad, Omer Elfaroug Amin Mohammed, Vinod Kumar Singhal, Hassan Yousuf Bilal

**Affiliations:** 1 Department of Orthopedic Surgery, PRIME Hospital, Dubai, ARE; 2 Department of Surgery, Gladstone Hospital, Perth, AUS; 3 Department of General Surgery, PRIME Hospital, Dubai, ARE

**Keywords:** acupressure pc6, general surgery, observational study, ondansetron, postoperative nausea and vomiting

## Abstract

Objectives: The objective of this study was to compare the efficacy of ondansetron and acupressure at pericardium 6 (PC6 or Neiguan) points in managing postoperative nausea and vomiting (PONV).

Methodology: This observational study was conducted in the Department of Orthopedics at Prime Hospital, Dubai, UAE, between 2019 and 2020. Data collection comprised two main components. The first involved recording demographic details, including patients’ age, gender, and education level. The second component assessed the severity of nausea and vomiting using a validated visual analog scale (VAS), ranging from 0 to 10, where 0 indicates no nausea, 1-3 represent mild nausea, 4-7 indicat moderate nausea, and 8-10 reflect severe nausea. Symptom severity and frequency were documented at one, three, and seven hours postoperatively.

Results: The mean age of the acupressure group (Group A) was 38.92 ± 12.11 years, while that of the ondansetron group (Group B) was 35.91 ± 12.7 years. A statistically significant difference was observed between the groups regarding nausea severity and episode frequency; however, no significant difference was found in vomiting events. Acupressure demonstrated superior effectiveness in reducing both the severity and frequency of postoperative nausea, as well as in minimizing vomiting episodes, compared to ondansetron.

Conclusion: The findings suggest that acupressure at the PC6 point is more effective than ondansetron in managing PONV, particularly in reducing symptom duration and intensity.

## Introduction

Postoperative nausea and vomiting (PONV) is a common and significant complication that can adversely affect a patient’s recovery and overall health [1]. These adverse effects warrant a nursing diagnosis. The incidence of PONV has historically been reported in 20%-30% of patients, varying depending on the type of surgery and patient-specific factors [2]. Surgeries performed under spinal anesthesia are particularly associated with a higher incidence of PONV due to psychological factors, intraoperative hypotension, and opioid use [3]. Several studies have reported a high prevalence of PONV (70-80%) in obese patients, with a notably higher incidence in females [1-5]. Severe PONV can lead to complications such as bleeding, wound dehiscence, dehydration, and pulmonary issues, ultimately resulting in prolonged hospital stays [4].

Various antiemetics have been used to manage PONV, with ondansetron initially introduced for chemotherapy-induced vomiting. Due to its high efficacy, it has also become widely used for PONV [5]. However, despite its effectiveness, ondansetron is associated with adverse effects, including diarrhea, headaches, and alterations in hepatic enzyme levels [6]. Consequently, non-invasive alternatives are increasingly being explored. Relaxation techniques such as acupressure and acupuncture have shown promise in alleviating PONV. Multiple studies have demonstrated the effectiveness of acupressure in managing both PONV and postoperative pain [7]. Specifically, acupressure has been effective in relieving symptoms following gynecological surgeries, laparoscopic cholecystectomy, and appendectomy [8-11].

This study aims to evaluate the efficacy of acupressure at the pericardium 6 (PC6) point and compare it with the effectiveness of ondansetron in managing PONV. The objective is to assess the success of both ondansetron and PC6 acupressure in reducing PONV.

## Materials and methods

Study design

This prospective observational study was conducted in the Department of Orthopedics at PRIME Hospital, Dubai, UAE, between 2019 and 2020. The primary objective was to evaluate and compare the effectiveness of two antiemetic approaches - acupressure wristbands and ondansetron injections - on the incidence and severity of PONV in patients undergoing elective orthopedic surgery under spinal anesthesia.

The study was approved by the Institutional Review Board of Prime Hospital (protocol no. IRB/00450/18). According to the ethical principles outlined in the Declaration of Helsinki, all participants received information about the study objectives and procedures and provided written informed consent before enrollment.

Sample size calculation

The sample size was calculated using the general formula for comparing two means:

n = [2 × (Zα/2 + Zβ)² × σ²] ÷ Δ²

Where:

Zα/2 = 1.96 (for 95% confidence level)

Zβ = 0.84 (for 80% power)

σ = standard deviation

Δ = minimum clinically significant difference

Based on these parameters, a minimum of 64 participants (32 per group) was required. To account for possible dropouts, a total of 70 patients were enrolled.

Participant selection

Patients were selected using consecutive sampling from individuals scheduled for elective orthopedic surgeries under spinal anesthesia at PRIME Hospital between 2019 and 2020. Eligibility was assessed during the pre-anesthesia evaluation conducted by the orthopedic and anesthesiology teams. Patients were included if they were male or female, aged between 15 and 60, and scheduled for elective orthopedic procedures under spinal anesthesia. Written informed consent was obtained from all participants. Patients were excluded if they had any pre-existing condition affecting the PC6 acupoint, such as skin lesions or trauma in the area. Additional exclusion criteria included comorbidities associated with acute or chronic nausea and vomiting, such as gastrointestinal or auditory disorders, a history of neurological illness, current or prior alcohol or opioid use, previous experience with acupressure therapy, or pregnancy. Patients were also excluded if they developed a postoperative temperature of 38°C [12] or higher or required additional antiemetic medication postoperatively. All eligible and consenting patients were enrolled until the calculated sample size was reached.

Surgical procedures

All patients underwent elective orthopedic surgery, which included internal fixation for fractures, arthroscopic procedures, and other standard orthopedic interventions. Each method was performed under spinal anesthesia according to hospital protocols. The duration of surgeries ranged between 45 and 90 minutes.

Treatment group details

Participants received acupressure wristbands or ondansetron injections as part of routine clinical care and were grouped accordingly. Group A included patients who used acupressure wristbands, while Group B included those who received ondansetron.

**Figure 1:**
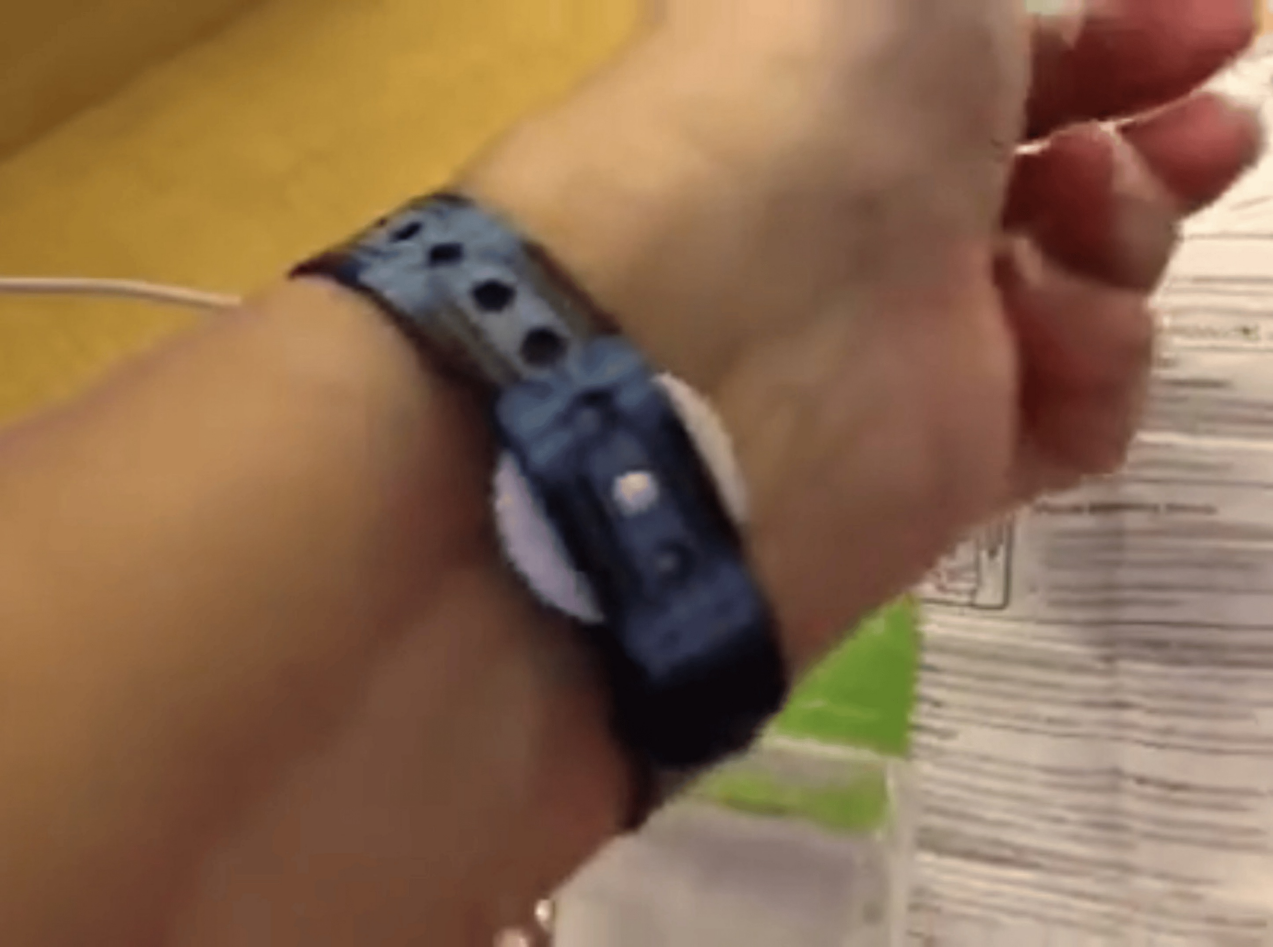
Points of pericardium (PC)

**Figure 2:**
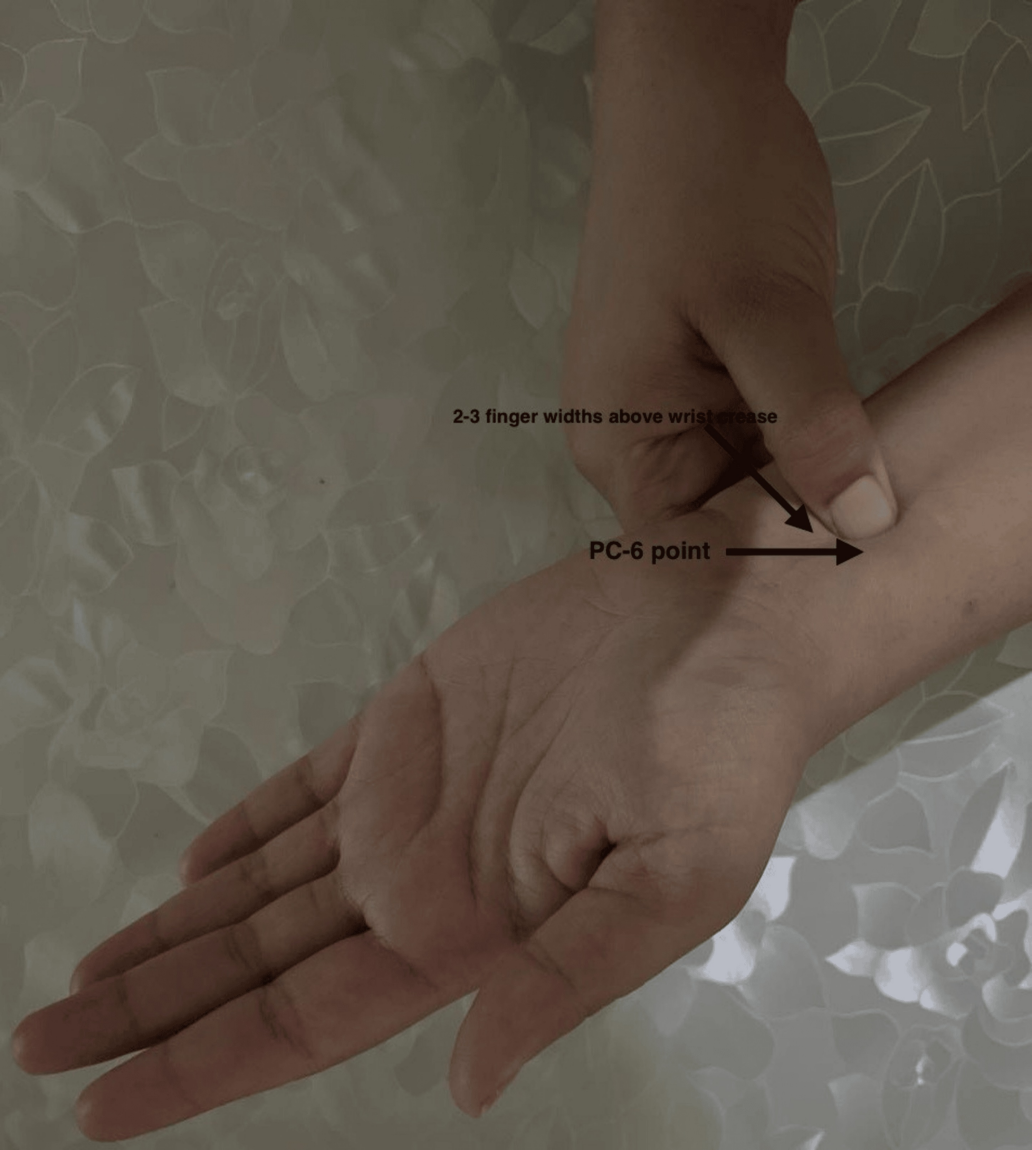
PsiBand on the patient's wrist

Group A participants received PsiBand wristbands (FDA-approved) (Figure 1), which were applied bilaterally at the PC6 (Neiguan) acupoint - located approximately 2-3 finger-widths above the wrist crease, between the tendons of palmaris longus and flexor carpi radialis. The application followed the standard three-finger method, with trained staff ensuring proper placement without compromising radial artery blood flow or venous return. The wristbands were adjusted periodically to maintain effectiveness and patient comfort (Figure 2).

Group B participants received ondansetron as part of the standard postoperative care protocol. The drug was administered intravenously at a dose of 0.1 mg/kg once daily for three consecutive days. Adjustments to the dose were made based on clinical judgment and individual patient response.

Data collection

Data collection involved two components. First, demographic data, including age, gender, and education level, were recorded. Second, the severity of nausea and vomiting was assessed using a validated visual analog scale (VAS) ranging from 0 to 10. Severity was classified as follows: 0 = no nausea, 1-3 = mild, 4-7 = moderate, and 8-10 = severe. Assessments were conducted at one, three, and seven hours after surgery. Both symptom intensity and frequency were documented during these intervals.

Statistical analysis

Data were analyzed using Statistical Product and Service Solutions (SPSS, version 23; IBM SPSS Statistics for Windows, Armonk, NY). Descriptive statistics were used to summarize the data, including means and standard deviations (SD) for continuous variables and frequencies with percentages for categorical variables. Comparative analysis was conducted using the chi-square test for categorical variables. For continuous variables, particularly the VAS scores, independent samples t-tests were performed to compare means between groups, as the data were approximately normally distributed. A p-value of less than 0.05 was considered statistically significant.

## Results

Table 1 presents the demographic characteristics of participants in both treatment groups. The age distribution was comparable between Group A (acupressure) and Group B (ondansetron), with no statistically significant difference (p = 0.416). Participants were divided into three age categories, and although a slightly higher proportion of younger individuals (15-30 years) was observed in the ondansetron group, these differences were not statistically significant. The sex distribution was also balanced, with a marginally higher proportion of females in both groups. Chi-square analysis confirmed no considerable variation in sex distribution (p = 0.632), indicating demographic comparability between the groups at baseline.

**Table 1:** Demographic characteristics of the patients

Variables	Group A (acupressure) (N =35)	Group B (ondensteron) (N=35)	Chi-square value	p value
Age			1.75	0.416
46-60 years	11 (31.4%)	10 (28.5%)		
31-45 years	15 (42.8%)	11 (31.4%)		
15-30 years	9 (25.7%)	14 (40%)		
Sex			0.23	0.632
Female	20 (57.1%)	17 (48.5%)		
Male	15 (42.8%)	18 (51.4%)		

Before surgery, none of the participants in either group reported any nausea. In Group A (acupressure), eight (22.9%) had mild, 20 (57.1%) moderate, and seven (20%) severe nausea. In Group B (ondansetron), 18 (51.4%) had mild, 12 (34.3%) moderate, and five (14.3%) severe nausea. At one hour postoperatively, four (11.4%) in Group A and two (5.7%) in Group B reported no nausea, with most patients experiencing mild nausea. However, severe nausea was present only in Group B (two, 5.7%). By three hours, improvement was more marked in the acupressure group, with 12 (34.3%) experiencing no nausea and none reporting severe symptoms, while the ondansetron group still had eight (22.9%) in the severe category. At seven hours, 24 (68.6%) in the acupressure group reported no nausea, and none had severe symptoms, compared to zero (0%) reporting no nausea in the ondansetron group and six (17.1%) still experiencing severe nausea (Table 2).

**Table 2:** Nausea severity based on the visual analog scale (VAS) category

Time interval	No nausea		Mild (1-3)		Moderate (4-7)		Severe (8-10)	
	Group A	Group B	Group A	Group B	Group A	Group B	Group A	Group B
Before surgery	0 (0%)	0 (0%)	8 (22.9%)	18 (51.4%)	20 (57.1%)	12 (34.3%)	7 (20%)	5 (14.3%)
1 hour postoperatively	4 (11.4%)	2 (5.7%)	23 (65.7%)	22 (62.9%)	8 (22.9%)	9 (25.7%)	0 (0%)	2 (5.7%)
3 hours postoperatively	12 (34.3%)	3 (8.6%)	21 (60%)	12 (34.3%)	2 (5.7%)	12 (34.3%)	0 (0%)	8 (22.9%)
7 hours postoperatively	24 (68.6%)	0 (0%)	10 (28.6%)	17 (48.6%)	1 (2.9%)	12 (34.3%)	0 (0%)	6 (17.1%)

Table 3 presents a comparative analysis of nausea severity and the number of nausea episodes between the acupressure group (Group A) and the ondansetron group (Group B) at different postoperative intervals. Preoperatively, Group A reported significantly higher nausea severity than Group B (p < 0.001). Although no significant difference was noted at one hour postoperatively (p = 0.29), Group A exhibited a marked reduction in nausea severity at both three and seven hours following surgery (p < 0.001), indicating improved outcomes over time. Similarly, there was no significant difference in the frequency of nausea episodes before surgery (p = 0.55). However, Group A reported significantly fewer episodes at one, three, and seven hours postoperatively compared to Group B, with all p-values less than 0.001. These findings suggest that acupressure was more effective than ondansetron in reducing the severity and frequency of postoperative nausea, particularly during the later recovery period.

**Table 3:** Comparison of nausea episodes and severity between the acupressure and ondansetron groups * represents a significant p-value

Duration of surgery	Group A	Group B	t-test	p-value
Nausea severity				
Before surgery	3.60 ± 0.95	1.86 ± 0.81	8.24	<0.001^*^
1 hour postoperatively	2.74 ± 0.85	2.97 ± 0.98	-1.04	0.29
3 hours postoperatively	1.71 ± 0.67	3 ± 0.97	-6.74	<0.001^*^
7 hours postoperatively	1.11 ± 0.32	6.54 ± 1.2	-25.8	<0.001^*^
Nausea episodes				
Before surgery	3.43 ± 0.95	3.26 ± 1.42	0.58	0.55
1 hour postoperatively	2.62 ± 0.69	4.26 ± 1.12	-7.37	<0.001^*^
3 hours postoperatively	1.82 ± 0.66	5.57 ± 0.78	-21.71	<0.001^*^
7 hours postoperatively	1.23 ± 0.43	6.66 ± 0.80	-35.3	<0.001^*^

Table 4 compares the incidence of vomiting events between the two groups across the same time intervals. Vomiting frequencies were comparable before surgery (p = 0.434). However, from the first postoperative hour onward, Group A consistently experienced fewer vomiting episodes than Group B. The difference was statistically significant at one hour (p = 0.0004) and even more pronounced at three and seven hours (p < 0.0001 and p < 0.00001, respectively). These results further support the greater effectiveness of acupressure in controlling postoperative vomiting over time.

**Table 4:** Comparison of vomiting events between the acupressure and ondansetron groups * presents a significant p-value

Duration	Group A	Group B	t-test	p-value
Before surgery	1.23 ± 0.43	1.26 ± 0.46	0.778	0.434
1 hour postoperatively	1.03 ± 0.17	1.34 ± 0.48	-3.72	0.0004^*^
3 hours postoperatively	0.46 ± 0.41	2.17 ± 0.12	-22.6	<0.0001^*^
7 hours postoperatively	0.26 ± 0.13	2.94 ± 0.68	-19.49	<0.00001^*^

Table 5 shows the side effects reported in both treatment groups. Patients who received ondansetron experienced significantly more side effects, including headache, nervousness, fever, and injection site inflammation, with all differences showing statistical significance (p < 0.05). In contrast, the acupressure group reported fewer side effects overall. Minor issues such as mild skin marks and wrist discomfort were observed only in the acupressure group, but these were not statistically significant. These findings suggest that acupressure is effective and better tolerated than ondansetron.

**Table 5:** Side effects among both groups * represents a significant p-value

Side effects	Group A	Group B	Chi-square	p-value
Headache	2 (5.7%)	9 (25.7%)	5.01	0.025^*^
Nervousness	1 (2.9%)	6 (17.1%)	4.12	0.042^*^
Fever	0 (0%)	4 (11.4%)	4.21	0.040^*^
Inflammation	0 (0%)	5 (14.3%)	5.38	0.020^*^
Skin marks	3 (8.6%)	0 (0%)	3.21	0.073
Discomfort	2 (5.7%)	0 (0%)	2.06	0.151

## Discussion

PONV remains a frequent and distressing complication following surgery, adversely affecting patient comfort and the quality of postoperative recovery. Although multiple studies have demonstrated the antiemetic effects of acupressure in patients undergoing general anesthesia [13-18], the present study specifically focused on its efficacy in patients receiving spinal anesthesia for lower limb procedures (e.g., fracture repair, joint surgery, and orthopedic shoe-related interventions) and lower abdominal surgeries (including hernia repair and appendectomy). The mean duration of surgical procedures in this study ranged between 45 and 90 minutes, and all patients were monitored according to standard perioperative protocols.

The findings of our study indicate that PC6 acupressure significantly reduced the incidence of nausea at one hour postoperatively compared to ondansetron. Additionally, the frequency of severe nausea cases at three and seven hours post-anesthesia was lower in the acupressure group. These results are consistent with previous literature supporting the use of acupressure in various surgical contexts [13-15]. Similarly, through a meta-analysis, Faheem et al. [13] confirmed that acupressure significantly reduces early postoperative nausea scores in general and regional anesthesia settings, further supporting the method's broader applicability. Kamali et al. [14] likewise demonstrated that acupressure improves comfort and reduces reliance on antiemetic medication in ambulatory lower abdominal procedures, reinforcing the potential for PC6 stimulation to alleviate PONV in patients not receiving general anesthesia.

Contrasting outcomes have been reported by Zhao et al. [15] and Ongel et al. [16], who found minimal efficacy of acupressure in laparoscopic abdominal surgeries. This discrepancy may be attributable to methodological limitations, including inaccurate PC6 point localization, suboptimal pressure intensity, and insufficient follow-up duration. The current study gave careful attention to accurate point identification and pressure application, which may account for the more favorable outcomes. Additionally, the broader age range and inclusion of older adults in this study may have influenced results, as prior studies have often involved younger populations and smaller sample sizes.

Ondansetron remains a widely accepted first-line antiemetic agent, with reported efficacy rates approaching 90%. However, in this study, adverse effects - such as headache, nervousness, fever, and swelling - were more frequently observed in the ondansetron group than in the acupressure group. These findings are supported by previous reports from Fu et al. [17] and Larki et al. [18], who documented reduced tolerability of ondansetron due to its side effect profile.

Importantly, reduced postoperative vomiting was observed in the acupressure group, corroborating the findings of Eslami et al. [9], who demonstrated a significant decrease in vomiting frequency with PC6 stimulation in regional anesthesia contexts. Notably, several studies have proposed that PC6 acupressure may exert a more substantial effect on nausea than on vomiting. Eslami et al. [9] highlighted this distinction, positing that acupressure more effectively modulates nausea-related neural pathways, while its influence on the vomiting reflex may be comparatively limited. These mechanistic nuances may account for variations in reported outcomes and underscore the complex pathophysiology of PONV.

An additional feature of this study was its focus on patients under spinal anesthesia, in which individuals remained conscious throughout the surgical procedure. This permitted the observation of psychological influences on nausea. Variability in anxiety levels among conscious patients likely contributes to differential nausea severity. Salamah et al. [19] recently demonstrated that acupressure may alleviate physiological stress by reducing stress biomarkers and promoting serotonin release. It offers a potential mechanistic explanation for its effectiveness in conscious surgical patients. The age-related variability observed in our study further underscores the importance of investigating the influence of physiological and psychological factors on the efficacy of acupressure.

Alternative explanations rooted in traditional medical theories have also been proposed. For instance, Echeverria-Villalobos et al. [20] discussed the role of acupressure in modulating the flow of vital energy (“Qi”), thereby promoting gastric motility and psychological calm. Such mechanisms offer a complementary framework for understanding the clinical benefits of PC6 acupressure.

Given its safety, affordability, and favorable patient acceptance, this study's findings support the incorporation of PC6 acupressure as a potential first-line or adjunctive intervention for the prevention of PONV in patients undergoing spinal anesthesia for orthopedic and lower abdominal surgery. Nonetheless, larger multicenter randomized trials with standardized acupressure protocols and long-term follow-up are warranted to establish definitive clinical recommendations.

Limitations

Since spinal anesthesia was used in our study, patients remained conscious during surgery, and their psychological states may have influenced the outcomes. Additionally, age-related variations may have introduced potential bias. Therefore, further large-scale studies are recommended to evaluate the long-term effectiveness of acupressure in managing PONV.

## Conclusions

The present study demonstrates that acupressure at the P6 acupoint is a safe and effective method for reducing PONV in patients undergoing general surgery. The acupressure and ondansetron groups were demographically comparable at baseline, allowing for a reliable comparative analysis. Although ondansetron was effective during the early postoperative period, acupressure resulted in significantly better outcomes in controlling nausea and vomiting at three and seven hours postoperatively. Patients in the acupressure group experienced fewer and less severe episodes of PONV over time, indicating a more sustained antiemetic effect. These findings suggest that acupressure may serve as a practical, non-pharmacological alternative or adjunct to conventional antiemetic therapies. However, further large-scale, multicenter studies involving diverse patient populations and extended follow-up durations are recommended to validate these results and further explore the long-term efficacy, optimal timing, and standardized application techniques of acupressure in various surgical contexts.
